# Spiritual well-being, caregiver burden, depression, and quality of life in parents of children with spinal muscular atrophy: A cross-sectional study

**DOI:** 10.1097/MD.0000000000049156

**Published:** 2026-06-19

**Authors:** Neslihan Cansel, Bilge Özgör, Gül Yücel

**Affiliations:** a Department of Psychiatry, Faculty of Medicine, İnönü University, Malatya, Türkiye; b Department of Pediatrics, Division of Pediatric Neurology, Faculty of Medicine, İnönü University, Malatya, Türkiye.

**Keywords:** caregiver burden, caregivers, depression, quality of life, spinal muscular atrophy, spiritual well-being

## Abstract

Spinal muscular atrophy (SMA) imposes substantial physical and emotional burdens on families, particularly on primary caregivers. Although spiritual well-being has been linked to psychological resilience in chronic diseases, its relationship with caregiver burden, depressive symptoms, and quality of life in caregivers of children with SMA remains insufficiently studied. This study investigated these associations in parents of children with SMA. This cross-sectional online survey included 51 parents of children with genetically confirmed SMA (types I–III). Participants completed questionnaires including sociodemographic and clinical characteristics, the Spiritual Well-Being Scale, Zarit Burden Interview, Patient Health Questionnaire-9 (PHQ-9), and WHOQOL-8.Tr. Correlation and exploratory regression analyses were performed to examine relationships between study variables. The mean Spiritual Well-Being Scale, Zarit Burden Interview, Patient Health Questionnaire-9, and WHOQOL-8.Tr scores were 119.2 ± 15.5, 49.5 ± 12.8, 11.2 ± 5.0, and 25.4 ± 5.8, respectively. Spiritual well-being was negatively correlated with caregiver burden (*r* = −0.492, *P* < .001) and depressive symptoms (*r* = −0.380, *P* = .002), and positively correlated with quality of life (*r* = 0.486, *P* < .001). Caregivers of children requiring medical devices had higher burden and depressive symptom scores and lower spiritual well-being scores (*P* < .05). Employment and higher income levels were associated with better quality of life and lower caregiver burden (*P* < .05). Caregivers using prayer as a coping strategy reported higher spiritual well-being scores (*P* = .007). In exploratory regression analyses, higher spiritual well-being scores were associated with lower caregiver burden and depressive symptoms and with higher quality-of-life scores. Higher spiritual well-being was associated with lower caregiver burden and depressive symptoms and with better quality of life among parents of children with SMA. Given the cross-sectional design and limited sample size, these findings should be interpreted cautiously. Further longitudinal and multicenter studies are needed to better clarify the role of spiritual well-being in caregiver outcomes among families affected by SMA.

## 1. Introduction

Spinal muscular atrophy (SMA) is one of the most severe pediatric neuromuscular disorders, characterized by progressive muscle weakness, respiratory compromise, and high caregiving demands.^[1,2]^ Although advances in pharmacological and rehabilitative treatments have improved prognosis, the daily care of children with SMA continues to impose substantial physical, emotional, and social burdens on families, particularly on primary caregivers. Parents frequently report stress, anxiety, and depressive symptoms, which not only affect their own well-being but may also influence the child’s clinical outcomes and quality of life.^[3–5]^ In addition, caregiver outcomes may be influenced by multiple factors, including disease severity, functional dependency, socioeconomic conditions, and caregiving intensity.

Caregiver burden in chronic and rare diseases has been widely studied, and increasing attention has been paid to the role of spiritual well-being in caregiver adaptation and psychological coping. Spirituality provides a sense of meaning, hope, and resilience in the face of prolonged stress, and recent studies have reported associations between higher spiritual well-being and lower caregiver burden and depressive symptoms.^[6,7]^ In parents caring for children with rare disorders, religiosity and spirituality are frequently cited as coping resources that mitigate burnout and enhance perceived quality of life.^[8,9]^

Moreover, spirituality often interacts with contextual variables such as social support, economic resources, cultural values, and caregiving-related stressors. For instance, Taher et al^[10]^ found that both spiritual well-being and perceived social support were significantly associated with caregiving burden among families of children with disabilities.^[10]^ This is particularly relevant in societies where religious and spiritual practices are deeply integrated into daily life, as in Türkiye, where prayer and faith are commonly used coping mechanisms among caregivers.

Despite these findings, research focusing specifically on caregivers of children with SMA remains scarce. Existing evidence highlights the multidimensional challenges faced by these families,^[11]^ but little is known about how spirituality, caregiver burden, depressive symptoms, and quality of life interact in this unique population. Addressing this gap is crucial, as SMA imposes long-term caregiving responsibilities that may amplify psychological distress while simultaneously fostering reliance on spiritual coping strategies.^[4,12,13]^

Therefore, the present study aims to investigate the cross-sectional relationships between spiritual well-being, caregiver burden, depressive symptoms, and quality of life among primary caregivers of children with SMA. By integrating current evidence and focusing on this understudied population, the study seeks to contribute novel insights to both clinical care and psychosocial support strategies for families affected by SMA.

## 2. Materials and methods

### 2.1. Study design and ethical approval

This study was conducted as cross-sectional observational research between January and May 2025 at İnönü University Faculty of Medicine, Türkiye. Ethical approval was obtained from the Non-Interventional Clinical Research Ethics Committee of İnönü University, Faculty of Medicine (Approval No: E-27942812-100-539288, Date: January 09, 2025). All study procedures complied with the principles of the Declaration of Helsinki. Informed consent was obtained electronically from all participants prior to data collection. The study was reported in accordance with the Strengthening the Reporting of Observational Studies in Epidemiology guidelines.

### 2.2. Participants

The study population consisted of parents who were the primary caregivers of children with genetically confirmed SMA (types I–III). A total of 51 participants were included in the final analysis. Eligibility criteria required being at least 18 years of age, serving as the main caregiver, the ability to read and understand Turkish, and voluntary participation. Exclusion criteria were the presence of severe psychiatric illness or active psychotic episode, major life crisis during the study period, inability to complete the questionnaire independently, or having >1 caregiver from the same family to avoid duplicate data.

**Figure 1.**
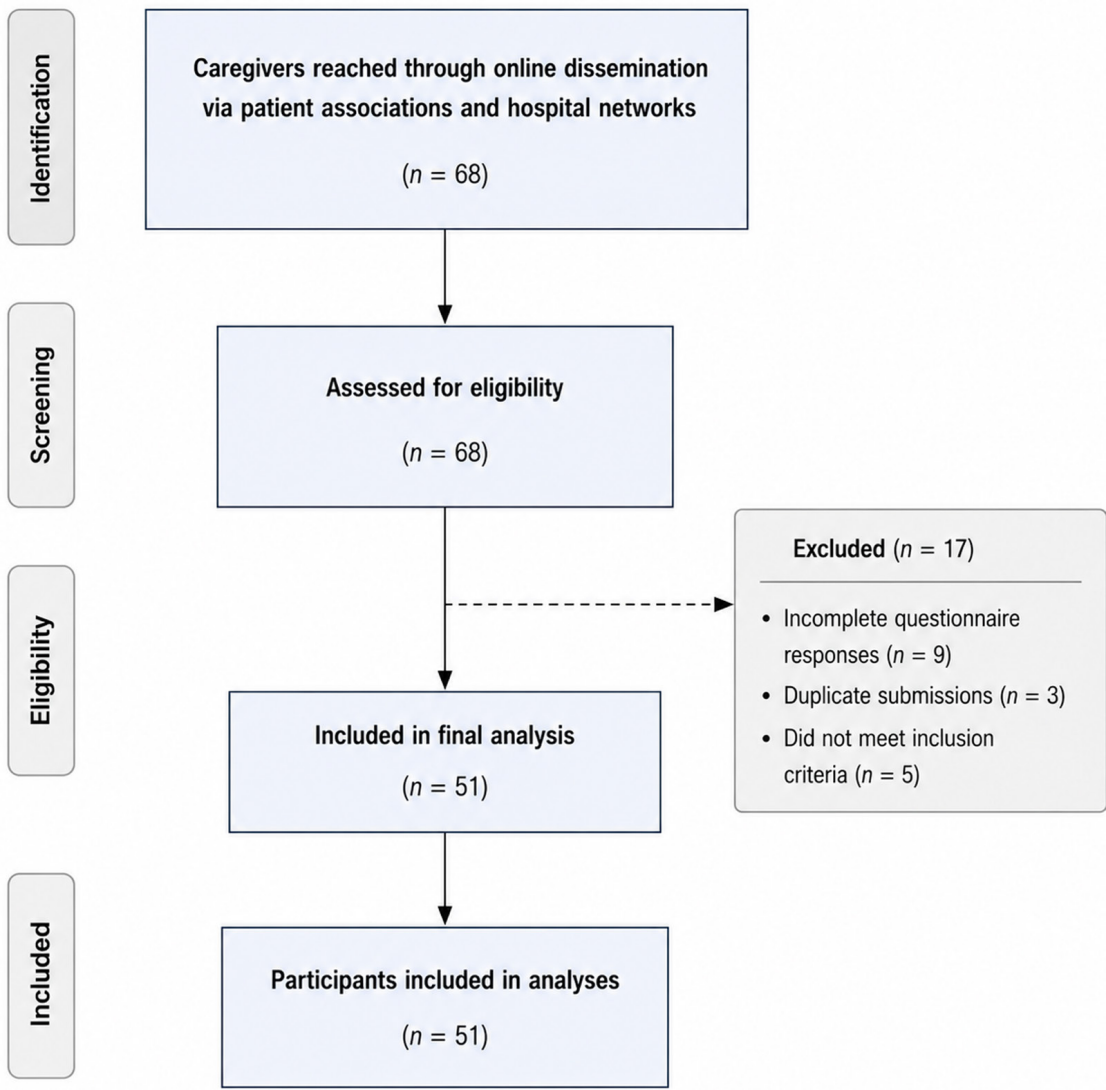
Flow diagram illustrating participant recruitment, eligibility assessment, exclusion process, and final study inclusion.

Participants were recruited online via patient associations and hospital networks using a convenience sampling approach. The questionnaire link was distributed through caregiver support groups and institutional communication channels. Participation was voluntary and anonymous, and no financial incentive was provided. A participant flow diagram describing the recruitment and inclusion process was provided in Figure 1. All responses were collected anonymously and stored in a password-protected database. Questionnaires with substantial missing or incomplete responses were excluded prior to analysis.

### 2.3. Data collection

Sociodemographic data included caregiver age, gender, marital status, education level, monthly income, employment status, family structure, relationship to the child, caregiving experience, daily caregiving duration, and the presence of additional caregiving support. Information regarding the number of siblings in the household and hospitalization history of the child was also collected. Child-related data included SMA type, age, disease duration, and medical device requirements. Where available, caregivers were additionally asked about treatment-related characteristics and caregiving support conditions. The online questionnaire was designed to be completed in approximately 20 to 25 minutes, and all responses were stored in an anonymized database.

### 2.4. Measures

Spiritual Well-Being Scale (SWBS, Turkish version): The SWBS consists of 21 items grouped into 3 subdimensions: transcendence, harmony, and meaning. Items are rated on a 5-point Likert scale, with total scores ranging from 21 to 141. Higher scores indicate greater levels of spiritual well-being. The Turkish adaptation has been validated and shown to have high internal consistency.^[14]^

Zarit Burden Interview (ZBI, Turkish version): The ZBI is a 22-item self-report scale that assesses subjective caregiver burden. Each item is scored on a 5-point Likert scale, and total scores range from 0 to 88. Higher scores indicate greater perceived burden. The Turkish validation study confirmed strong psychometric reliability.^[15]^

Patient Health Questionnaire-9 (PHQ-9, Turkish version): The PHQ-9 consists of 9 items assessing depressive symptoms experienced in the past 2 weeks. Each item is scored from 0 (“not at all”) to 3 (“nearly every day”), yielding a total score range of 0 to 27. In addition to continuous score analysis, depressive symptom severity categories were descriptively evaluated according to established PHQ-9 cutoff values. The Turkish version has been validated with acceptable sensitivity and specificity.^[16]^

World Health Organization Quality of Life-8 (Turkish version) (WHOQOL-8.Tr [European Health Interview Survey – Quality of Life 8-item Index]): The European Health Interview Survey – Quality of Life 8-item Index is a short version of the WHOQOL instrument adapted into Turkish, containing 8 items covering general health and overall quality of life domains. Each item is scored on a 5-point scale, and higher total scores indicate better quality of life. The Turkish version has been validated and widely used in chronic disease populations.^[17]^

### 2.5. Statistical analysis

All statistical analyses were performed using IBM SPSS Statistics version 21.0 (IBM Corp.). Continuous variables were expressed as mean ± standard deviation, and categorical variables were presented as frequencies (n, %). The normality of distributions was assessed using the Shapiro–Wilk test. For 2-group comparisons, Student *t* test was used when assumptions were met, and the Mann–Whitney *U* test when assumptions were not satisfied. For comparisons involving more than 2 groups, 1-way analysis of variance or the Kruskal–Wallis test was applied, with Bonferroni post hoc correction for multiple comparisons. Correlations between continuous variables were analyzed using Spearman rank correlation coefficient. Effect sizes were interpreted according to conventional thresholds: very weak (<0.20), weak (0.20–0.39), moderate (0.40–0.59), strong (0.60–0.79), and very strong (≥0.80). A 2-tailed *P* value <.05 was considered statistically significant.

Exploratory regression analyses were performed to further examine associations between spiritual well-being and caregiver-related outcomes. Given the relatively limited sample size, regression findings were interpreted cautiously and considered hypothesis-generating rather than confirmatory. Clinically relevant variables, including caregiver age, caregiver gender, and child SMA type, were considered during model construction where applicable. A complete-case analysis approach was used, and no imputation procedure was applied for missing data. Sensitivity analyses were additionally performed by comparing main correlation findings across selected clinical and sociodemographic subgroups.

Sample size was determined a priori using G*Power version 3.1 (Heinrich Heine University Düsseldorf, Düsseldorf, Germany), based on correlation analysis with an assumed medium effect size (*r* = 0.30), α = 0.05, and power = 0.80. The required minimum sample was 47 participants; thus, the inclusion of 51 participants provided adequate statistical power for the primary correlation analyses of the study.^[6]^

## 3. Results

The study included 51 caregivers. The mean caregiver age was 37.4 ± 6.3 years. Most participants were female caregivers (31, 60.9%), mothers serving as the primary caregiver (29, 57.8%), married (48, 93.8%), and living in nuclear family structures (41, 79.7%). Among the children, 28 (54.9%) had SMA type I, and 18 (35.3%) required medical device support (Table 1).

**Table 1:** Sociodemographic and clinical characteristics of caregivers and children (n = 51).

Variable	n (%) or Mean ± SD
Caregiver age (yr)	37.4 ± 6.3
Female caregiver	31 (60.9)
Mother as primary caregiver	29 (57.8)
Married	48 (93.8)
Nuclear family structure	41 (79.7)
Currently employed	20 (39.1)
Monthly income: 23,001–73,000 Turkish Lira	22 (43.1)
Child with SMA type I	28 (54.9)
Medical device requirement	18 (35.3)

SD = standard deviation, SMA = spinal muscular atrophy.

The mean SWBS total score was 119.2 ± 15.5, ZBI total score was 49.5 ± 12.8, PHQ-9 total score was 11.2 ± 5.0, and WHOQOL-8 total score was 25.4 ± 5.8 (Table 2).

**Table 2:** Descriptive statistics of study scales.

Scale	Mean ± SD	Median (Min–Max)
SWBS total	119.2 ± 15.5	122 (64–141)
ZBI total	49.5 ± 12.8	47.5 (28–81)
PHQ-9 total	11.2 ± 5.0	10 (4–27)
WHOQOL-8 total	25.4 ± 5.8	26 (11–37)

PHQ-9 = Patient Health Questionnaire-9, SWBS = Spiritual Well-Being Scale, WHOQOL-8 = World Health Organization Quality of Life-8, ZBI = Zarit Burden Interview.

Spearman correlation analysis demonstrated a negative correlation between SWBS and both ZBI (*r* = −0.492, *P* < .001) and PHQ-9 scores (*r* = −0.380, *P* = .002). In contrast, SWBS was positively correlated with WHOQOL-8 scores (*r* = 0.486, *P* < .001). Longer physiotherapy duration was associated with higher SWBS scores (*r* = 0.298, *P* = .017), whereas longer nutrition duration was associated with higher ZBI scores (*r* = 0.338, *P* = .006) (Table 3).

**Table 3:** Correlations between study scales and clinical variables.

Variables	*r*	*P* value
SWBS – ZBI	–0.492	<.001
SWBS – PHQ-9	–0.380	.002
SWBS – WHOQOL-8	0.486	<.001
Physiotherapy duration – SWBS	0.298	.017
Nutrition duration – ZBI	0.338	.006

PHQ-9 = Patient Health Questionnaire-9, SWBS = Spiritual Well-Being Scale, WHOQOL-8 = World Health Organization Quality of Life-8, ZBI = Zarit Burden Interview.

Group comparisons demonstrated significant differences according to sociodemographic and clinical characteristics. Caregivers with prior caregiving experience had higher ZBI subdimension 3 scores (*P* = .038). Families without additional caregiving support showed higher SWBS scores (*P* = .021). Medical device requirement was associated with higher PHQ-9 and ZBI scores and lower SWBS scores (*P* < .05). Employed caregivers reported higher WHOQOL-8 subdimension 2 scores (*P* = .021). Higher income levels were associated with lower ZBI total scores and higher WHOQOL-8 scores (*P* = .032 and *P* = .024, respectively). In addition, caregivers who reported prayer as a coping strategy had higher SWBS transcendence subscale scores (*P* = .007) (Table 4).

**Table 4:** Group comparisons of study scales according to sociodemographic and clinical variables.

Variable	Scale outcome	*P* value
Caregiving experience (yes vs no)	ZBI subdimension 3	.038
Additional caregiving support (no vs yes)	SWBS total	.021
Medical device requirement (yes vs no)	SWBS, PHQ-9, ZBI total	<.05
Employment (yes vs no)	WHOQOL-8 subdimension 2	.021
Monthly income (>73,001 TL vs <23,000 TL)	ZBI total, WHOQOL-8 total	.032; .024
Coping by prayer (yes vs other)	SWBS subdimension 1	.007

PHQ-9 = Patient Health Questionnaire-9, SWBS = Spiritual Well-Being Scale, WHOQOL-8 = World Health Organization Quality of Life-8, ZBI = Zarit Burden Interview.

Exploratory regression analyses examining associations between spiritual well-being and caregiver-related outcomes are presented in Table 5. Higher total SWBS scores were associated with lower ZBI total scores (β = −0.482, *t* = −3.92, *P* < .001, *R*^2^ = 0.26) and lower PHQ-9 total scores (β = −0.371, *t* = −3.15, *P* = .003, *R*^2^ = 0.19), while higher SWBS scores were associated with higher WHOQOL-8 total scores (β = 0.486, *t* = 4.10, *P* < .001, *R*^2^ = 0.28).

**Table 5:** Exploratory regression analyses examining associations between spiritual well-being and caregiver-related outcomes.

SWBS variable	Outcome variable	β (standardized)	*t*	*P* value	Model *R*^2^
SWBS total score	ZBI total score (caregiver burden)	−0.482	−3.92	<.001	0.26
SWBS total score	PHQ-9 total score (depressive symptoms)	−0.371	−3.15	.003	0.19
SWBS total score	WHOQOL-8 total score (quality of life)	+0.486	4.10	<.001	0.28
SWBS – transcendence subscale	ZBI total score	−0.417	−3.21	.002	0.21
SWBS – harmony subscale	PHQ-9 total score	−0.329	−2.87	.006	0.17
SWBS – meaning subscale	WHOQOL-8 total score	+0.455	3.95	<.001	0.25

Regression findings should be interpreted cautiously due to the limited sample size and exploratory nature of the analyses.

PHQ-9 = Patient Health Questionnaire-9, SWBS = Spiritual Well-Being Scale, WHOQOL-8 = World Health Organization Quality of Life-8, ZBI = Zarit Burden Interview.

When SWBS subdimensions were analyzed separately, the Transcendence subscale was associated with lower caregiver burden scores (β = −0.417, *t* = −3.21, *P* = .002, *R*^2^ = 0.21), the Harmony subscale was associated with lower depressive symptom scores (β = −0.329, *t* = −2.87, *P* = .006, *R*^2^ = 0.17), and the Meaning subscale was associated with higher quality-of-life scores (β = 0.455, *t* = 3.95, *P* < .001, *R*^2^ = 0.25). All regression models met assumptions of normality, independence, and homoscedasticity, and no substantial multicollinearity was detected (VIF < 2.0 for all predictors).

## 4. Discussion

This study investigated the relationships between spiritual well-being, depression, caregiver burden, and quality of life among parents of children with spinal muscular atrophy. The findings demonstrated that spiritual well-being was negatively associated with depressive symptoms and caregiver burden, while being positively associated with quality of life. These findings suggest that spiritual well-being may be related to more favorable psychological and quality-of-life outcomes among caregivers of children with SMA. In the context of SMA, a rare and progressive neuromuscular disease, spirituality may represent an important coping resource that helps parents attribute meaning and maintain emotional balance while managing long-term caregiving responsibilities.

The negative association between spiritual well-being and caregiver burden observed in this study aligns with prior research in various chronic illness contexts. Mirhosseini et al^[6]^ showed that higher levels of spiritual well-being were linked to lower burden among stroke caregivers.^[6]^ Similarly, Vaishnav et al^[18]^ reported that spirituality reduced caregiver burden and enhanced psychological resilience.^[18]^ The current results extend these findings to parents of children with SMA, although the cross-sectional design precludes conclusions regarding causality or directionality of these relationships.^[19]^

The inverse association between spirituality and depression in our sample also reflects prior findings. Gonzalez et al^[20]^ highlighted that parents of children with cancer who had higher spiritual orientation reported significantly fewer depressive symptoms and lower burnout.^[20]^ Spirituality may contribute to adaptive coping processes through meaning-making, hope, and emotional support mechanisms. However, these associations should be interpreted cautiously, as depressive symptoms and spiritual well-being may influence each other bidirectionally. Our findings are generally consistent with previous studies conducted in caregivers of children with chronic neurological and life-limiting conditions.^[21,22]^

The positive relationship between spiritual well-being and quality of life in this study is noteworthy. Domaradzki et al^[9]^ reported that religious beliefs improved quality of life in parents of children with rare diseases, which is consistent with our results.^[9]^ The beneficial associations observed between spirituality and quality of life may arise not only from individual coping strategies but also from perceived social support and the attribution of meaning to the caregiving process. Nevertheless, quality of life in caregivers is likely influenced by multiple interacting clinical, psychological, and socioeconomic factors beyond spirituality alone.^[23,24]^

The exploratory regression analyses presented in Table 5 further demonstrated statistically significant associations between spiritual well-being and caregiver-related outcomes among parents of children with SMA. However, given the relatively limited sample size and the observational cross-sectional design, these findings should be considered exploratory and hypothesis-generating rather than confirmatory. In line with previous studies involving caregivers of individuals with chronic and life-limitingconditions,^[6,7,18]^ our findings demonstrated that higher levels of spiritual well-being independently predicted lower caregiver burden and depressive symptoms, as well as higher quality of life. Notably, the standardized coefficients indicated a moderate-to-strong effect size (β range: 0.37–0.49), underscoring that spirituality is not merely a passive coping resource but an active determinant of well-being in this population.

Among the subdimensions, meaning showed the strongest association with quality of life, suggesting that the ability to find purpose and significance in the caregiving experience may mitigate emotional exhaustion and enhance perceived fulfillment. The transcendence and harmony dimensions were also significant, reflecting how faith-based connectedness and inner balance can reduce stress-related symptoms and depressive affect. These findings are consistent with recent studies reporting that spiritual and existential resources predict better mental health and resilience in caregivers of children with neurological and genetic disorders.^[9,10,23]^

Although several sociodemographic variables were considered during analysis, residual confounding cannot be excluded. Factors such as disease severity, functional dependency, treatment-related characteristics, and caregiver psychosocial background may also have influenced the observed relationships. Therefore, the present findings should not be interpreted as evidence of an independent causal effect of spiritual well-being on caregiver outcomes. Rather, the results suggest that spirituality may represent 1 component within the broader psychosocial context of caregiving experiences in SMA.

Sociodemographic and clinical findings further highlight the complex interplay between contextual factors and caregiver outcomes. Employed parents reported a higher quality of life, while those with higher income showed lower burden and better quality of life, suggesting that socioeconomic security plays a pivotal role in reducing stress and improving well-being.^[25]^ The need for medical devices was associated with higher depressive symptoms, higher burden, and lower spiritual well-being, underscoring the impact of disease severity and technological care demands on parental outcomes. Prior caregiving experience was related to higher burden, which may reflect cumulative strain over time. Interestingly, parents without additional caregiving support reported higher spiritual well-being, possibly indicating a greater reliance on personal spiritual resources when external support is lacking.^[26,27]^ Finally, those who used prayer as a coping strategy reported significantly higher spiritual well-being, a finding that is particularly meaningful in societies where religious and spiritual practices play a central cultural role.^[22]^

Taken together, these findings contribute to the growing body of literature examining spirituality and caregiver well-being in chronic pediatric conditions. While numerous studies have focused on caregivers of cancer or neurodegenerative disorders, research specifically addressing parents of children with SMA remains limited. Accordingly, the present findings may provide preliminary insight into the potential relationships between spirituality and caregiver outcomes in this understudied population.

## 5. Limitations

The study, however, is not without limitations. First, the sample size was relatively modest, with only 51 parents included, which limits the generalizability of the results. In particular, the relatively limited sample size should be considered when interpreting the exploratory regression analyses, as the statistical power may have been insufficient for more comprehensive multivariable modeling. Larger, multicenter studies are needed to validate and expand upon these findings.

Second, the cross-sectional design precludes causal inferences; it remains unclear whether higher spiritual well-being contributes to lower caregiver burden and depressive symptoms or whether caregivers with lower psychological distress report better spiritual well-being. Accordingly, the observed associations should not be interpreted as directional or causal relationships.

Third, participants were recruited through online dissemination via patient associations and hospital networks using a convenience sampling approach. This recruitment strategy may have introduced selection bias, as caregivers with greater digital access, stronger social engagement, or higher motivation to participate may have been overrepresented. In addition, response rates and characteristics of nonparticipants could not be fully assessed, limiting evaluation of sample representativeness.

Fourth, all data were collected through self-report questionnaires administered concurrently, which may be subject to social desirability bias, recall bias, and subjective interpretation. No objective clinical or physiological measures of caregiver stress or child functional status were included to corroborate questionnaire-based findings.

Fifth, although several sociodemographic and clinical variables were considered, residual confounding cannot be excluded. Potentially relevant factors such as treatment modality, detailed functional dependency, hospitalization burden, caregiver psychiatric history, and broader psychosocial support systems were not comprehensively evaluated and may have influenced caregiver outcomes.

Sixth, the study employed a limited set of measures; other relevant dimensions of spirituality, such as organized religious activity, community participation, existential meaning-making, and longitudinal changes in coping patterns, were not assessed. Furthermore, the cross-sectional assessment could not capture temporal changes in spirituality, caregiver burden, or mental health status over time.

Finally, as the study was conducted in Türkiye within a specific cultural and social context, cultural factors related to spirituality, religion, and caregiving practices may limit the applicability of the findings to other populations. These limitations should be carefully considered when interpreting the results, and future longitudinal, multicenter studies with larger and more diverse samples are warranted.

## 6. Conclusion

In conclusion, this study demonstrated significant associations between spiritual well-being, caregiver burden, depressive symptoms, and quality of life among parents of children with SMA. Higher spiritual well-being scores were associated with lower caregiver burden and depressive symptoms and with better quality of life. In addition, sociodemographic and clinical factors, including income level, employment status, medical device requirement, and coping strategies, were also associated with caregiver outcomes.

Given the cross-sectional design and methodological limitations of the study, these findings should be interpreted cautiously and should not be considered evidence of causal relationships. Nevertheless, the results suggest that spiritual well-being may represent a relevant psychosocial dimension within the broader caregiving experience of families affected by SMA. Future longitudinal and multicenter studies are needed to further clarify the role of spirituality and other psychosocial factors in caregiver adaptation and well-being in this population.

## Author contributions

**Conceptualization:** Neslihan Cansel, Bilge Özgör, Gül Yücel.

**Data curation:** Neslihan Cansel, Bilge Özgör, Gül Yücel.

**Formal analysis:** Neslihan Cansel, Bilge Özgör, Gül Yücel.

**Funding acquisition:** Neslihan Cansel, Bilge Özgör, Gül Yücel.

**Investigation:** Neslihan Cansel, Bilge Özgör, Gül Yücel.

**Methodology:** Neslihan Cansel, Bilge Özgör, Gül Yücel.

**Project administration:** Neslihan Cansel, Bilge Özgör, Gül Yücel.

**Resources:** Neslihan Cansel, Bilge Özgör, Gül Yücel.

**Software:** Neslihan Cansel, Bilge Özgör, Gül Yücel.

**Supervision:** Neslihan Cansel, Bilge Özgör, Gül Yücel.

**Validation:** Neslihan Cansel, Bilge Özgör, Gül Yücel.

**Visualization:** Neslihan Cansel, Bilge Özgör, Gül Yücel.

**Writing – original draft:** Neslihan Cansel, Bilge Özgör, Gül Yücel.

**Writing – review & editing:** Bilge Özgör, Gül Yücel.
